# Supplementary material for: Mutations in PCYT2 disrupt etherlipid biosynthesis and cause a complex hereditary spastic paraplegia
Source: Brain. 2019 Oct 22;142(11):3382–97. doi: 10.1093/brain/awz291 (PMC6821184; doi:10.1093/brain/awz291)
Supplement: awz291_Supplementary_Data [file awz291_supplementary_data.zip › awz291-Suppl_data/Supplementary_Data4.zip!/Supplementary information 4 - plasma lipidomics.zip!/Plasma lipidomics/Control vs Patient/Statistical plots and data/Heatmap sorted on FDR.pdf]

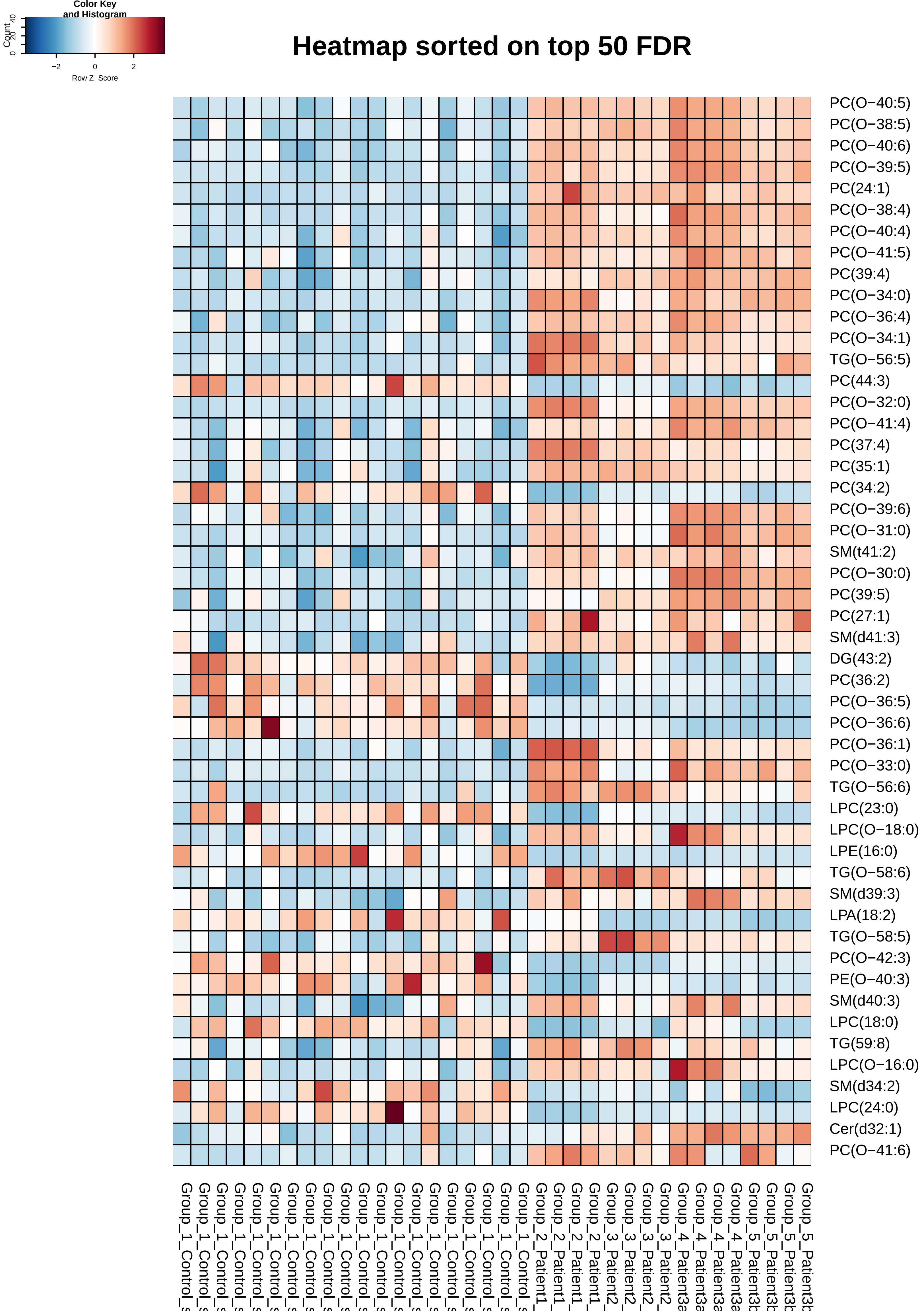
Color Key
and Histogram
Count
Row Z−Score
Heatmap sorted on top 50 FDR
PC(O−40:5)
PC(O−38:5)
PC(O−40:6)
PC(O−39:5)
PC(24:1)
PC(O−38:4)
PC(O−40:4)
PC(O−41:5)
PC(39:4)
PC(O−34:0)
PC(O−36:4)
PC(O−34:1)
TG(O−56:5)
PC(44:3)
PC(O−32:0)
PC(O−41:4)
PC(37:4)
PC(35:1)
PC(34:2)
PC(O−39:6)
PC(O−31:0)
SM(t41:2)
PC(O−30:0)
PC(39:5)
PC(27:1)
SM(d41:3)
DG(43:2)
PC(36:2)
PC(O−36:5)
PC(O−36:6)
PC(O−36:1)
PC(O−33:0)
TG(O−56:6)
LPC(23:0)
LPC(O−18:0)
LPE(16:0)
TG(O−58:6)
SM(d39:3)
LPA(18:2)
TG(O−58:5)
PC(O−42:3)
PE(O−40:3)
SM(d40:3)
LPC(18:0)
TG(59:8)
LPC(O−16:0)
SM(d34:2)
LPC(24:0)
Cer(d32:1)
PC(O−41:6)
Group_1_Control_s
Group_2_Patient1
Group_3_Patient2
Group_4_Patient3a
Group_5_Patient3b
